# Exploiting the peptidoglycan-binding motif, LysM, for medical and industrial applications

**DOI:** 10.1007/s00253-014-5633-7

**Published:** 2014-03-21

**Authors:** Ganesh Ram R. Visweswaran, Kees Leenhouts, Maarten van Roosmalen, Jan Kok, Girbe Buist

**Affiliations:** 1 Department of Molecular Genetics, Groningen Biomolecular Sciences and Biotechnology Institute (GBB), University of Groningen, Nijenborgh 7, 9747 AG Groningen, The Netherlands; 2 Department of Medical Microbiology, University of Groningen, University Medical Center Groningen, Hanzeplein 1, P.O. Box 30001, 9700 RB Groningen, The Netherlands; 3 Mucosis BV, L.J. Zielstraweg 1, 9713 GX Groningen, The Netherlands; 4 Present Address: Department of Medical Biology, University of Groningen, University Medical Center Groningen, Hanzeplein 1, P.O. Box 30001, 9700 RB Groningen, The Netherlands

**Keywords:** LysM, Noncovalent peptidoglycan binding, Vaccine, Cell immobilization, Protein display, Microbe detection

## Abstract

The lysin motif (LysM) was first identified by Garvey et al. in 1986 and, in subsequent studies, has been shown to bind noncovalently to peptidoglycan and chitin by interacting with *N-*acetylglucosamine moieties. The LysM sequence is present singly or repeatedly in a large number of proteins of prokaryotes and eukaryotes. Since the mid-1990s, domains containing one or more of these LysM sequences originating from different LysM-containing proteins have been examined for purely scientific reasons as well as for their possible use in various medical and industrial applications. These studies range from detecting localized binding of LysM-containing proteins onto bacteria to actual bacterial cell surface analysis. On a more applied level, the possibilities of employing the LysM domains for cell immobilization, for the display of peptides, proteins, or enzymes on (bacterial) surfaces as well as their utility in the development of novel vaccines have been scrutinized. To serve these purposes, the chimeric proteins containing one or more of the LysM sequences have been produced and isolated from various prokaryotic and eukaryotic expression hosts. This review gives a succinct overview of the characteristics of the LysM domain and of current developments in its application potential.

## Introduction

### Cell wall binding domains

The cell envelope of Gram-positive bacteria consists of a cytoplasmic membrane and a cell wall. Besides protecting and maintaining cell integrity, the cell wall is important for interactions of the cell with its external milieu. The major component of the Gram-positive cell wall is peptidoglycan (PG), which consists of glycan chains cross-linked through short peptides and interpeptide bridges. PG can be divided into multiple types on the basis of variations in the glycan strands, the peptide stem, or in the position or composition and degree of the interpeptide bridge formation. Additional variations of the PG can occur through amidation, hydroxylation, acetylation, or attachment of amino acids or other groups (Vollmer et al. 2008b). Components that are incorporated in or attached to the PG include teichoic acids, carbohydrates, and proteins. Teichoic acids are involved in binding of cations and proteins and in determining the electrochemical properties of the cell wall. Wall teichoic acids are attached to PG, while lipoteichoic acids (LTA) are linked to the cytoplasmic membrane of the cell (Silhavy et al. 2010). Polysaccharides can be covalently bound or loosely associated to the wall. Differences between species are found in the sugar monomers, the type of linkage between them, their substitutions, and whether the polysaccharides are branched or not, all resulting in variations of the physicochemical properties of the cell wall.

Proteins present in the cell wall are either attached to the cytoplasmic membrane, to the PG, or to other cell wall components. On average, around 5 to 10 % of the proteins produced by a Gram-positive bacterium are translocated across the cytoplasmic membrane (Schneewind and Missiakas 2012). Some of these proteins are fully secreted, while others remain attached to the membrane after translocation due to the presence of a membrane-spanning domain or a lipo-modification. PG-binding proteins are either covalently attached through via a so-called LPxTG domain and the action of a sortase or will be noncovalently immobilized by interaction with PG, teichoic acids, polyphosphates, or carbohydrates via anionic interactions (Schneewind and Missiakas 2012). The LPxTG domain that is used to covalently anchor bacterial proteins to PG in the cell wall has been extensively investigated, and this knowledge has been used in various applications such as protein modification, vaccine development, and enzyme- or whole-cell immobilization (Leenhouts et al. 1999; Proft 2010). A general characteristic of the proteins that bind noncovalently to the cell wall is the presence of one or more so-called cell wall-binding domains (CWBDs) that bind to a specific ligand of the cell wall. Often, a separate domain constitutes the actual active site of the protein. Either the N- or C-terminal part of such a cell wall-bound protein could be displayed on the bacterial cell surface. The CWBD is involved in anchoring of the protein to PG only, or its function is to properly position the catalytic domain of the protein in the vicinity of its substrate as has been shown for some phage lysins (Low et al. 2011)*.* The noncovalent binding domains have been suggested to enable a local increase in the protein/enzyme concentration in the cell wall. The modular composition of the cell wall-anchored proteins of Gram-positive bacteria is the subject of ongoing investigations, while several proteomic approaches are also being used to identify and characterize these proteins and to identify the parts that are surface-exposed (Desvaux et al. 2006; Dieye et al. 2010; Dreisbach et al. 2011; Vollmer et al. 2008a). In addition, CWBDs that bind proteins to bacterial cell walls have been employed in various (medical) applications. Among these are PG- and choline-binding domains, teichoic acid-binding domains, domains of S-layer proteins, and poly-sugar-binding domains (Schmelcher et al. 2012). The domains that have been identified to specifically and noncovalently bind to PG are listed in Table 1.

**Table 1:** Overview of noncovalent peptidoglycan binding domains used in various applications

PFAM	Ligand	Application(s)	Reference
LysM (PF01476)	*N*-acetylglucosamine	See Fig. 1	This review
SH3b (PF08460)	Pentaglycine bridge	Detection of *S. aureus*, lysis of specific bacteria	Gründling and Schneewind (2006), Lu et al. (2006), Schmelcher et al. (2012)
WxL domain (PF13731)	PG	Display of nuclease	Brinster et al. (2007)
PG-binding domain type 1 (PF01471)	PG	–	Ghuysen et al. (1994), Li et al. (2011)
CWBDs of *Listeria* phage endolysins	Likely PG	Detection of *Listeria*, immobilization of target cells	Schmelcher et al. (2012), Tolba et al. (2012)
CWBD	Amidated d-Asp cross-bridge	Detection of specific bacteria	Regulski et al. (2013)

### Peptidoglycan-specific binding domains

In 1994, Ghuysen et al. showed that PG hydrolases with different specificities expressed by *Clostridia* and *Bacillus* strains contain N- or C-terminal repeated sequences involved in cell wall binding. Many years later, this PG-binding domain type 1 (PF01471) has been shown to specifically bind PG although the ligand had not been identified yet (Li et al. 2011). Brinster et al. (2007) showed that the C-terminal WxL domain is present in 27 *Enterococcus faecalis* V583 proteins of which two (EF0392 and EF2686) specifically bind PG. Fusion of this domain with nuclease (Nuc) of *Staphylococcus aureus*, an enzyme that is normally secreted, resulted in binding of the fusion protein to purified PG from *Bacillus subtilis* and whole cells of *E. faecalis*, *B. subtilis*, *Streptococcus agalactiae*, *Lactobacillus johnsonii*, *Staphylococcus aureus*, and *Listeria innocua* when it was added from the outside. Also for this domain, the specific ligand has not been identified yet. Gründling and Schneewind (2006) showed that a fusion of the C-terminal cell wall-targeting (SH3b) domain of lysostaphin with green fluorescent protein (GFP) bound to *S. aureus* cells and purified PG. The SH3b domain specifically binds to the penta-glycine cross-bridge in *S. aureus* PG as was revealed by examining various *S. aureus* cell wall synthesis mutant. Lu et al. (2006) solved the structure of this domain and showed that it belongs to the SH3b domain family, the prokaryotic counterpart of the eukaryotic family of SH3 domains. The SH3b domain is present in many phage endolysins and may be involved in binding of the endolysin to different pathogens with subsequent lysis of the bacteria (Schmelcher et al. 2012).

Fusions of CWBDs of endolysins and different fluorescent proteins have been used for the specific detection and serotyping of *Listeria monocytogenes* or *Bacillus anthracis* cells (Tolba et al. 2012). Such fusion proteins were also used as biomarkers for the detection of Gram-negative bacteria upon prior permeabilization of the outer membrane of the cells. Using electrochemical impedance spectroscopy, the endolysin-CWBD500 was immobilized on a gold screen-printed electrode, which was subsequently used to detect up to 1.1 × 10^5^ and 10^4^ CFU ml^−1^ *Listeria* cells in milk and pure cultures, respectively (Tolba et al. 2012). Recently, the CWBDs of *Lactobacillus casei* endolysins Lc-Lys and Lc-Lys-2 have been shown to bind PG specifically at the amidated d-Asp cross-bridge (Regulski et al. 2013). This domain was used to target *Enterococcus faecium* cells and was suggested to have the potential to identify bacterial species with a d-Asp cross-bridge within a microbial community.

Garvey et al. (1986) were the first to describe the lysin motif (LysM), which is present twice within the C-terminus of the lysozyme of *Bacillus* phage Φ29. Using *N*-acetylglucosamine oligomers, Ohnuma et al. (2008) showed that the LysM domain specifically binds to *N*-acetylglucosamine moieties. Of the above listed PG-specific binding domains, the LysM domain has been studied in most detail and has been studied in different potential applications. This review summarizes the latest results obtained in the characterization of this very interesting protein domain and subsequently focuses on the various medical and industrial applications of the LysM domain.

## Characteristics of and variations in LysM domains

**Fig. 1:**
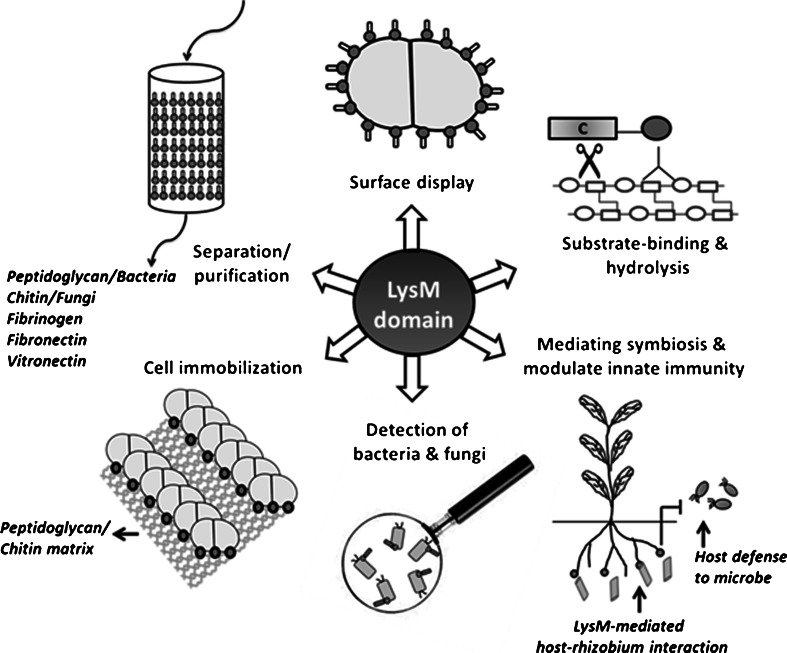
Schematic representation of molecular functions and applications of LysM domains. The LysM domains are depicted as *dark filled circles* (). A typical bacterial example (*L. lactis*) is shown (). The catalytic domain of a cell wall hydrolase is indicated as “C”. *Lollipops* () represent LysM domains fused to antigen/protein (for surface display) or to a matrix (for screening/purification)

The LysM domain is a very widespread CWBD. It consists of a repetition of a small motif, the LysM sequence (Bateman and Bycroft 2000; Buist et al. 2008). A single copy of this motif typically contains from 44 to 65 amino acid residues. It is represented in the Pfam database (PF01476) by more than 27,000 sequences from over 4,500 species of prokaryotic, eukaryotic, and viral origin (http://pfam.sanger.ac.uk/family/PF01476). Multiple LysM sequences in a protein are often separated by small Ser-, Thr-, and Asn-rich intervening sequences. The number of LysM sequences in a domain varies from 1 to up to 12; the domain is found mainly at the N- or C-termini of proteins and less frequently in the central region of some LysM-containing proteins (Table 2, Buist et al. 2008). It has been shown that the LysM sequences specifically bind to the *N*-acetylglucosamine moieties of PG or to *N*-acetylglucosamine-containing glycans such as chitin fragments, nodulation, or mycorrhization factors (Gust et al. 2012). Hirschhausen and coworkers have shown that the LysM domain of Aaa, an autolysin/adhesion protein from *S. aureus*, binds to the plasma and extracellular matrix glycoproteins fibronectin, vitronectin, and fibrinogen (Hirschhausen et al. 2012). The authors speculate that the LysM domain binds to the carbohydrate moiety of these glycoproteins (Fig. 1).

**Table 2:** Overview of LysM-containing fusion proteins used for detection of bacteria, for cellular immobilization, or for display of enzymes/proteins on cell surfaces of Gram-positive bacteria

Species	LysM domain^a^	No. of LysMs^b^	Fusion or modification^c^	Produced in/bound to	Function/use^d^	Reference
*L. lactis* MG1363	Glucosaminidase (AcmA)	C-3	α-Amylase (*B. licheniformis*), TEM-β-lactamase (*E. coli*)	*L. lactis*	Surface display, purification	Leenhouts et al.(1999), Bosma et al. (2006)
*L. lactis* IL1403	Glucosaminidase (AcmA)	N-3, 2*(N-3), 3*N-3), 3*(C-3)	α-Amylase (*S. bovis* 148) (AmyA)	*L. lactis*	Surface display	Okano et al. (2008)
*L. lactis* IL1403	Glucosaminidase (AcmA)	C-3	Mutation of five potential N-glycosylation sites of LysM domain	*E. coli, Pichia pastoris*, *L. casei* NRRL B-441, *B. subtilis* 168, *E. coli* XL1-blue, *Saccharomyces cerevisiae* IFO0216	Substrate specificity	Tarahomjoo et al. (2008a)
*L. lactis* IL1403	Glucosaminidase (AcmA)	C-3	α-Amylase (*S. bovis* 148) (AmyA) and its starch binding domain	*E. coli*, *L. casei* NRRL B-441	Surface display	Tarahomjoo et al. (2008a, b, c)
*L. lactis* MG1363	Glucosaminidase (AcmA)	C-3	Cellulose-binding domain of XylA (*Cellvibrio japonicas*)	*L. lactis*, cellulose	Cell immobilization	Kyla¨-Nikkila et al. (2010)
*L. lactis* MG1363	Glucosaminidase (AcmA)	C-3	Chitin-binding domain (ChBD of chitinase A1) (*B. circulans*)	*L. lactis*, chitin	Cell immobilization	Şimşek et al. (2013)
*S. aureus*	CHAP (AAA)	N-3	6*His	*E. coli*, fibronectin, vitronectin, fibrinogen	Substrate specificity	Hirschhausen et al. (2012)
*S. aureus*	CHAP (Sle1), LytN (CHAP)	N-3, N-1	mCherry	*E. coli*, *S. aureus*	Detection	Frankel at al. (2012)
*L. lactis* MG1363	Glucosaminidase (AcmA)	N-3	Pseudomurein-binding domain (*Methanothermobacter thermautotrophicus*), GFP	*E. coli*, *L. lactis* (BLPs), shrimp shell chitin flakes, fungi	Detection, substrate specificity	Visweswaran et al. (2012)
*L. lactis* MG1363	Glucosaminidase (AcmA)	C-3 or C-2	Replacement of tryptophan by fluorescent analog	*L. lactis*, *L. lactis* (BLPs), PG from *Brevibacterium lactofermentum*	Detection	Petrović et al. (2012)
*L. lactis* MG1363	Glucosaminidase (AcmA)	C-3	c-myc	*L. lactis*	Substrate specificity	Andre et al. (2008, 2010), Tripathi et al. (2012)
*L. lactis* MG1363	Glucosaminidase (AcmD)	C-3	GFP	*E. coli*, *L. lactis* (BLPs)	Detection	Visweswaran et al. (2013)
*E. coli*	MltD	N-1	*p*-Cyanophenylalanine and tryptophan	Synthetic	Protein folding	Glasscock et al. (2008)
*B. thuringiensis* YBT-1520	Glucosaminidase (MbG) (WP_003257638)	2* (N-2)	Multicopper oxidase/laccase (WlacD), GFP	*B. thuringiensis*	Detection, surface display	Shao et al. (2012)
*B. subtilis*	d,l-Endopeptidases (LytE)	N-3	TEM-β-lactamase (*E. coli*)	*B. subtilis*	Surface display	Chen et al. (2008)
*L. plantarum*	muropeptidase (MurO)	N-2	β-Galactosidase, GFP	*E. coli*, *Bifidobacterium bifidium*	Detection, surface display	Xu et al. (2011)
*L. fermentum* bacteriophage ΦPYB5	Endolysin (Lyb5) (ABP88927)	C-3	GFPuv, β-galactosidase (*Paenibacillus*)	*E. coli*, *L. lactis*, *L. casei*, *L. brevis*, *L. plantarum*, *L. fermentum*, *L. delbrueckii*, *L. helveticus*, *S. thermophilus*	Surface display	Hu et al. (2010)
*L. lactis* MB191	Glucosaminidase (AcmA)	N-AcmA	Endo-beta-1, 3-1, 4-glucanase (*gls*) *B. subtilis* BF7658, GFP	*L. lactis* AS1.2829	Detection, surface display	Li et al. (2009)
*L. fermentum*	Aggregation-promoting factor (SEP) (AAS55430)	N-1	6*His	*L. fermentum*, *L. rhamnosus*, *L. lactis*	Surface display	Turner et al. (2004)
*L. lactis*	AcmA	C-1, C-3	B domain of staphylococcal protein A, TNF-α-binding affibody gene (*sdz-tnf*)	*L. lactis*	Surface display	Ravnikar et al. (2010)
*L. lactis*	AcmA	C-3	Receptor-binding domain F18 fimbrial adhesin FedF (*E. coli*)	*L. lactis* NZ9000∆*htrA*	Surface display	Lindholm et al. (2004)

^a^Origin of the LysM domain used in the fusion proteins

^b^Location and number of the LysM sequences within the fusion protein:“C”, C-terminal; “N”, amino-terminal

^c^Enzymatic specificity and origin of the protein

^d^Function or use of the fusions as indicated in Fig. 1

One LysM sequence has a βααβ secondary structure with the two helices packing onto the same side of an antiparallel β sheet (Bateman and Bycroft 2000). All conserved hydrophobic residues form part of a continuous hydrophobic core and are mostly involved in the packing of the two helices onto the β sheet. Binding of the LysM sequence is mediated by a shallow groove on the surface of the motif formed by the two loops between each α helix and β strand. Binding results in the formation of several hydrogen bonds and is stabilized by van der Waals interactions (Liu et al. 2012; Sánchez-Vallet et al. 2013). Binding pockets for the *N*-acetyl group of the *N*-acetylglucosamine monomers have been identified in the LysM sequence, providing substrate specificity. A single LysM sequence binds, with affinity in the low micromolar range, a chain of up to five monosaccharides that exhibit an alternation of 180° flipping along the chain in fully extended conformation (Liu et al. 2012).

LysM-containing proteins can be found in most cellular compartments of prokaryotes and eukaryotes although the majority are secreted proteins, many are noncovalently bound to PG, while still others are inserted in or attached to the (outer-) membrane or have a cytosolic location (Buist et al. 2008; Gust et al. 2012). Many of the bacterial proteins with LysM domains are cell wall hydrolases. Most of those are PG hydrolases that contain a glucosaminidase, muramidase, amidase, or endopeptidase active site domain (Buist et al. 2008). The LysM domain in this group of proteins is required to bind the enzymes to PG and properly position the active site domain(s) toward their substrates. Thus, the local concentration of the enzymes is thought to be increased (Steen et al. 2003, 2005a). Certain LysM-containing plant proteins are receptors that mediate the recognition of different *N*-acetylglucosamine-containing ligands such as bacterial PG, fungal chitin, or rhizobacterial nodulation factors. These ligands contain important signatures for immune activation or establishment of beneficial plant-microbe symbiotic interactions (Gust et al. 2012). Mutation of the LysM receptor-like kinase LYK4 of *Arabidopsis* has been shown to enhance susceptibility to both a fungal and a bacterial pathogen; thus, this kinase improves the plant defense response against these pathogens (Wan et al. 2012). In contrast, it has been proposed that LysM-containing proteins in fungi may have a role in preventing their detection by the plants they invade. The chitin oligosaccharide breakdown products of the fungal cell walls are tightly bound by these fungal proteins thereby preventing them to act as trigger of an antifungal plant immune response (de Jonge and Thomma 2009). LysM domain-containing proteins are also common in animals. Only recently, their roles in these organisms are being investigated. The LysM-containing protein *Pc*LysM from the red swamp crayfish *Procambarus clarkia* was shown to confer antimicrobial immunity in the crayfish upon a challenge with the fish pathogen *Vibrio anguillarum* (Shi et al. 2013). Two distinct subfamilies of LysM proteins are highly conserved across vertebrates, namely LysMD and OXR (Laroche et al. 2013). A recent spatio-temporal gene expression analysis showed that the genes of both groups of proteins are expressed in the brain and nervous system during early stages of zebrafish larval development. No increased expression was detected in embryo models upon a challenge with bacterial pathogens, and the roles of these proteins in the fish remain elusive.

The attachment of bacterial secreted LysM proteins is generally restricted to site-specific locations on the bacterial cell envelope. Binding particularly takes place to sites of growth and cell division where newly synthesized PG is inserted into the cell wall (Buist et al. 2008). Interestingly, these sites of LysM binding are located near the location of protein secretion (Buist et al. 2006, 2008). Factors involved in localized binding have been investigated for the major autolysin AcmA of *Lactococcus lactis*, an *N*-acetylglucosaminidase containing a C-terminal LysM domain with three LysM sequences (Buist et al. 1995). The presence of cell wall constituents such as LTA hinders LysM binding to the cell wall and thereby likely directs AcmA to the site of cell separation. Removal of LTA from the PG layer of bacteria with trichloroacetic acid (TCA) enhances PG exposure and enables binding of a purified LysM domain-containing fusion protein to the entire cell surface (Andre et al. 2008; Steen et al. 2003; Vincent et al. 2013). Growth of *L. lactis* on galactose changes the LTA composition in the cell wall. Under these conditions, less AcmA is able to bind to the PG, resulting in decreased autolysis of the bacterial cells through AcmA activity (Steen et al. 2008). Furthermore, the presence of S-layer proteins impedes binding of AcmA (Steen et al. 2003), while O-acetylation and deacetylation of PG did not affect LysM-dependent binding of AcmA to PG (Meyrand et al. 2007; Veiga et al. 2007).

Recently, the LysM domains of LytN and Sle1 of *S. aureus* were shown to direct these hydrolases to the cross wall, the mid-cell compartment for PG synthesis (Frankel and Schneewind 2012). The binding at this location is also partly inhibited by PG modifications and wall teichoic acids. An *S. aureus* mutant that was affected in the binding of wall teichoic acids to the peptidoglycan lost the localized binding of both LysM-type murein hydrolases, which resulted in defects in cell shape and increased autolysis (Chan et al. 2013).

The isoelectric points of all known LysM-containing proteins ranges from 4 to 12, which was suggested to be important in adaptation to changing environmental pH values or to represent altered substrate-binding specificity (Buist et al. 2008). Indeed, Visweswaran et al. (2013) showed that the isoelectric point of the LysM domain is important in pH-dependent binding to PG of a GFP-LysM fusion protein. The *L. lactis* PG hydrolases AcmA and AcmD share a similar molecular architecture; the isoelectric points of their LysM domains, LysM_AcmA_ and LysM_AcmD_, are 10.0 and 4.2, respectively. Fusion proteins containing the LysM_AcmD_ domain could not bind to PG at a pH higher than its isoelectric point. A derivative of the *Lactobacillus fermentum* protein SEP carrying a His-tag between the signal peptide and the N-terminal LysM domain was secreted into the culture supernatant when expressed in *L. fermentum*, *Lactobacillus rhamnosus*, or *L. lactis* (Turner et al. 2004). In all three species, only a minor fraction of the protein ended up in the cell wall. The relatively low isoelectric point of 5.3 of LysM_SEP_ was suggested to explain the poor binding of the His-tagged fusion.

## Overexpression, isolation, and binding of LysM-containing proteins

Genetically incorporating a LysM domain in a protein of interest allows production, secretion, and ultimately easy isolation of the fusion protein from the culture medium of the genetically modified producer organism (GMO) (Leenhouts et al. 1999). Subsequently, the purified chimeric protein can be bound to and displayed on any (non-GMO) bacterium, spore, fungus, or other surface that contains an exposed binding ligand (PG or chitin) (van Roosmalen et al. 2006). LysM-containing proteins have been successfully expressed in different organisms. A LysM sequence alone, either as mono- or as multimer, or fused to other proteins, has been produced in Gram-negative (*Escherichia coli*) and several Gram-positive bacteria (*B. subtilis, B. thuringiensis*, *L. lactis*, *L. fermentum*, *L. rhamnosus*), in yeast (*Pichia pastoris*), insect cells (*Drosophila* sf21; Liu et al. 2012), and in mammalian cell lines (HEK293, CHO; Rigter et al. 2013) (Table 2).

A LysM domain provides an elegant way to isolate hybrid LysM-containing proteins. It was shown that by incubating a cell-free extract or a culture supernatant containing LysM_AcmA_ fusion proteins with a recipient “particle,” a PG-containing bacterial cell, these are simply isolated from the solution by centrifugation and washing of the particles (Bosma et al. 2006; van Roosmalen et al. 2006). As binding of the LysM_AcmA_ domain to bacterial cells is hindered by cell wall constituents (see above), this method is especially successful when these are first removed from the recipient cells by TCA treatment. For instance, bacterium-like particles (BLPs), formerly called Gram-positive enhancer matrix (GEM) (Bosma et al. 2006), are produced from acid- and heat-killed nonrecombinant *L. lactis* cells. They consist of spheres of PG lacking other intact cell wall components and intracellular material (Bosma et al. 2006; van Roosmalen et al. 2006). Binding of LysM_AcmA_ hybrid proteins to untreated lactic acid bacterial cells has also been used for protein purification, but their binding capacity was 10-fold lower compared to cells first treated with TCA (Bosma et al. 2006; Hu et al. 2010). Purification of LysM proteins can also be achieved in a single column-chromatography step, as has been shown for a protein with a LysM_AcmA_ tandem sequence using hydrophobic interaction chromatography (Petrović et al. 2012) and for a domain with three motifs using cation exchange chromatography (Steen et al. 2003; Bosma et al. 2006). Some LysM-containing proteins only carry a single motif, which seems enough for ligand binding (Downer et al. 2002; Frankel and Schneewind 2012; Longchamp et al. 1994; Percudani et al. 2005).

The formation of the LysM-PG bond is relatively fast and occurs with high affinity (Andre et al. 2008; Zeng et al. 2009). The optimal number of motifs required for maximum binding differs in the various proteins. For example, exogenously added AcmA derivatives carrying either two or four of the originally three LysM sequences bound to lactococcal cells, whereas the variants with no or only one motif did not (Steen et al. 2005a, b). Thus, at least two motifs seem to be required for proper cell wall binding of the LysM domain of AcmA when the enzyme is added to the cells from the outside (Steen et al. 2005a, b; Buist et al. 2008). Two LysM sequences are required for optimal binding of the endo-β-*N*-acetylglucosaminidase Mbg of *B. thuringiensis* (Shao et al. 2012). When expressed without the enzymatic domain, a single LysM_AcmA_ sequence is able to bind to PG; binding affinity steeply increases when two or three LysM sequences are present (Bosma et al. 2006).

## LysM fusions for binding studies, cell surface analysis, and microbe detection

LysM-containing proteins as well as chimeric fusions of LysM domains with various other proteins have been used to investigate their (localized) binding to bacterial cell surfaces (Table 2). A GFP fusion with the LysM-containing endo-β-*N*-acetylglucosaminidase (Mbg) from *B. thuringiensis* was successfully expressed and displayed on the whole cell surface of *B. thuringiensis* cells (Shao et al. 2012). Similarly, a fusion of GFP to a LysM-containing muropeptidase MurO from *L. plantarum* could be displayed on the surface of *L. plantarum* cells (Xu et al. 2011). Immunofluorescence detection of LysM proteins has been largely replaced by employing fusions of LysM domains with various fluorescent proteins in combination with fluorescence microscopy. The LysM_AcmA_ domain binds to different types of PG of *Bacillus*, *Lactococcus*, *Enterococcus*, *Streptococcus*, and *Lactobacillus* (Andre et al. 2008; Steen et al. 2003). The LysM domain of the endolysin Lyb5 of *L. fermentum*, C-terminally fused to GFP and a His-tag for easy purification, was isolated from *E. coli* and shown to attach to *L. lactis* and *L. casei* cells at the sites where newly synthesized PG is inserted into the cell wall (Hu et al. 2010). Furthermore, the fusion protein attached to the entire cell surfaces after TCA treatment of cells of *L. lactis*, various *Lactobacillus* species, and *Streptococcus thermophilus*. Binding of the fusion protein to the bacterial cells was 2-fold better near pH 11 than at pH 6 and was optimal using 0.5 M NaCl. Recently, using various techniques, the LysM_AcmA_ and LysM_AcmD_ domains were fused to GFP and shown to bind in a pH-dependent manner to the *L. lactis* cell wall (Visweswaran et al. 2012, 2013). Both fusion proteins bound to chitin and lactococcal BLPs, while LysM_AcmA_ additionally was shown to bind to fungal cell walls.

Fusions of mCherry to the C-termini of either the LysM or CHAP domains of LytN and to various LysM deletion mutants of the Sle1 protein of *S. aureus* revealed the crucial role of the LysM domain in localized binding of the two enzymes to the septum, thereby likely facilitating PG hydrolysis in daughter cell separation (Frankel and Schneewind 2012).

Petrović et al. (2012) biosynthetically labeled the tryptophan residues at different positions in LysM_AcmA_ with the tryptophan analogs 5-hydroxy tryptophan or 7-aza tryptophan and performed binding studies with the purified proteins using *L. lactis* BLPs and PG. Intrinsic fluorescence emitted by the tryptophan analogs after binding of the labeled proteins was detectable. One micromole of labeled LysM_AcmA_ domain was able to bind approximately 2 × 10^7^ BLPs showing that low levels of PG or small numbers of microorganisms in solution can be monitored using this approach (Petrović et al. 2012).

Apart from the above-mentioned applications for binding to and detection of microbial cells, LysM domains have been used to visualize the cell wall architecture of Gram-positive bacteria and for display of various heterologous proteins on bacterial cell surfaces. Atomic force microscopy (AFM) was recently performed to determine the nanoscale organization in living *L. lactis* cells of surface structures such as pili, polysaccharides, teichoic acids, and PG under physiological conditions (Andre et al. 2008, 2010; Tripathi et al. 2012). Using a LysM_AcmA_-coated AFM tip, the PG was shown to run parallel to the short axis of living cells of an *L. lactis* mutant that lacked cell wall polysaccharides (Andre et al. 2010).

## Cell immobilization

Immobilization, to entrap industrially relevant microorganisms in an inert matrix, is widely used for the industrial production of enzymes, proteins, antibiotics, and various other chemical compounds. Methods commonly used for immobilization of microbes include adsorption or attachment to a carrier, physical entrapment in polymers, microencapsulation, and membrane entrapment. In recent years, the industrial and medical applications of lactic acid bacteria (LAB) as biocatalysts have significantly increased. Certain species of LAB are mostly employed for the production of lantibiotics, β-galactosidase, nisin, lactic acid, and gamma-aminobutyric acid. LAB have also been used for the surface display of various heterologous proteins (Park et al. 2013; Vincent et al. 2013). LAB used in the food industry, such as *L. lactis*, are regarded as food-grade bacteria and might be good alternative biocatalysts as well. *L. lactis* cells have been immobilized on cellulose using a cellulose-binding domain fused to either the C-terminal LPxTG domain of the major proteinase PrtP (LPxTG_PrtP_; for covalent attachment of the fusion protein) or to LysM_AcmA_ (for noncovalent attachment of the protein). Immobilization of *L. lactis* cells to cellulose was achieved with both fusion proteins. Cells expressing the LPxTG_PrtP_-anchored fusion protein bound 34 % more efficiently than those with the LysM_AcmA_ domain (Kylä-Nikkilä et al. 2010). An *L. lactis* nisin-producing strain has been successfully immobilized to chitin flakes upon expression of the chitin-binding domain (ChBD) of chitinase A1 from *Bacillus circulans* fused to the LPxTG_PrtP_ domain or the LysM_AcmA_ domain (Şimşek et al. 2013). Immobilization efficiencies of cells expressing the ChBD-LPxTG_PrtP_ construct were significantly higher than those producing ChBD-LysM_AcmA_. Nevertheless, noncovalently bound proteins could be advantageous in some applications because the expressed and secreted protein can be isolated and added to non-GMO strains, e.g., to bind the latter to chitin or chitin flakes.

A fusion protein of LysM_AcmA_ with an archaeal pseudomurein-binding domain bound different substrates PG, chitin, and pseudomurein and could, for instance, be applied for the immobilization of whole cells of bacteria, fungi, or methanogenic archaea (Visweswaran et al. 2012). Immobilization of cells onto cellulose and chitin using LysM_AcmA_ fusion proteins has been shown to be feasible, and thus, more LysM fusion proteins in combination with different substrates should be tested to unveil the full range of possibilities for this type of application.

## Display of proteins, peptides, and enzymes

The LysM domains of various bacterial enzymes have been used to display chimeric (heterologous) fusion proteins onto cells of different bacterial species and various substrates (Table 2). The first successful example of the technology employed the C-terminal LysM_AcmA_ domain of *L. lactis* (Leenhouts et al. 1999). LysM_AcmA_ was fused to the C-terminus of *Bacillus licheniformis* α-amylase and *E. coli* TEM β-lactamase. Binding of the active heterologous chimeric proteins to *L. lactis* was achieved by incubating cells of an *L. lactis* AcmA deletion mutant with the culture supernatant of an *L. lactis* strain expressing and secreting either of the fusion proteins. Poquet et al. (2000) showed that AcmA is processed by the membrane-located protease HtrA, after its translocation across the cytoplasmic membrane. Zymographic analysis of the α-amylase and β-lactamase LysM_AcmA_ fusions showed that these are also subject to degradation by HtrA (Steen et al. 2003). Later it was shown that HtrA cleaves in the intervening sequences between the three LysM sequences of AcmA; no breakdown products were observed in an *htrA*-deficient strain (Steen et al. 2005a). d-Alanylation of LTA has been shown to result in increased proteolytic degradation of LysM proteins by HtrA (Steen et al. 2005b).

A similar fusion of *B. licheniformis* α-amylase with LysM_AcmA_ was used to show that an increase of 10- to 15-fold in enzyme activity could be obtained when the fusion protein was loaded onto *L. lactis* BLPs instead of *L. lactis* cells (Bosma et al. 2006). The acid treatment used to make BLPs also removes the membrane-located protease HtrA (Steen et al. 2005a, b). The nonproteolytic character of BLPs combined with their increased ability to bind LysM-containing proteins makes the use of these particles an attractive alternative to the use of whole cells in enzyme immobilization. Various combinations of enzymes can be immobilized onto BLPs at predetermined ratios in a controlled manner, as was shown by using different quantities of *L. lactis* supernatants containing the α-amylase-LysM_AcmA_ or TEM β-lactamase LysM_AcmA_ fusion proteins (Bosma et al. 2006). Okano et al. (2008) used both single and multiple N- and C-terminal fusions of LysM_AcmA_ with the α-amylase AmyA of *Streptococcus bovis* 148 to enhance the display of the enzymes on the cell surface of the producers of the fusion proteins, *L. lactis*, *L. plantarum*, or *L. casei*. The AmyA chimera with a C-terminal LysM_AcmA_ domain was enzymatically more active than the enzyme with the N-terminal fusion. Degradation products of the fusion proteins were observed, most probably as a consequence of proteolytic activities in the various bacterial species. Western hybridization and flow cytometry analyses in *L. lactis* IL1403 revealed that an increase in the number of LysM domains leads to an increase in the amount of cell wall-located fusion protein (Okano et al. 2008). A similar observation was made using an N-terminal fusion to GFP of the LysM domain of the glucosaminidase Mbg of *B. thuringiensis*, which is located at the N-terminal of this enzyme (Shao et al. 2012). Binding of LysM_Mbg_-GFP to cells of *B. thuringiensis* was observed when the most N-terminal LysM sequence of LysM_Mbg_ was used while no binding was obtained with the other of the two LysM_Mbg_ sequences. Binding improved considerably when the entire LysM_Mbg_ domain, with both motifs, was coupled two or three times to the N-terminus of GFP. Of a C-terminal fusion of LysM_AcmA_ with *S. bovis* α-amylase, only 9 % ended up in the soluble fraction of the producer in *E. coli* (Tarahomjoo et al. 2008a, b). Upon expressing the LysM_AcmA_ domain on its own in this host, 20 % of it ended up in the soluble fraction. The authors also showed that the yeast *P. pastoris* is an appropriate host for the expression of the *S. bovis* α-amylase-LysM_AcmA_ fusion protein.

The LysM_AcmA_ domain has been suggested to contain five potential N-glycosylation sites in the intervening sequences separating each motif (Tarahomjoo et al. 2008a, b). As glycosylation might interfere with cell-surface binding capacity, the amino acid sequences around these sites were changed by site-directed mutagenesis. The molecular weight of the mutated domain, expressed in *P. pastoris*, was higher than that of the wild-type domain, most likely as a consequence of O-glycosylation. The mutated domain bound to *L. casei* NRRL B-441 cells, albeit with a lesser affinity.

The LysM domain of the *B. subtilis* LytE cell wall hydrolase was fused either to the N- or C-terminus of *E. coli* TEM-β-lactamase (Chen et al. 2008). Both chimeric enzymes were biologically active and could be displayed onto the surface of *B. subtilis* cells. The maximum number of displayed molecules per cell (1.2 × 10^7^) was obtained for the purified N-terminal fusion with β-lactamase. Using immunofluorescence microscopy, it was shown that this fusion bound at high density to the entire *B. subtilis* cell surface.

Hu et al. (2010) produced a β-galactosidase from *Paenibacillus* sp. carrying the LysM domain of the endolysin Lyb5 of *L. fermentum* followed by a His-tag at its C-terminus in *E. coli*. The purified active fusion protein could be displayed on TCA-pretreated cells of *L. lactis* and on untreated cells of *L. casei*, *L. brevis*, or *L. plantarum*.

The examples presented above show that LysM domains can be used for protein/enzyme display. Recently, it has also been shown that full-length LysM-containing proteins can be used for the same purpose: An N-terminal fusion of *L. lactis* AcmA, with its C-terminal LysM domain, to GFP or the endo-β-1,3-1,4-glucanase from *B. subtilis* was functionally immobilized and displayed on the cell surface of the *L. lactis* producer cells (Li et al. 2009).

In conclusion, multiple enzymatically active LysM fusion proteins have been displayed successfully on the surfaces of various Gram-positive species. Both N- and C-terminal fusions have been used. Although degradation of the fusion proteins has been observed, this can be overcome by TCA treatment of the cell on which they are displayed. Display could in some cases be improved by increasing the number of LysM sequences in the fusion protein.

## Vaccines

One of the challenges in modern subunit vaccine development is to provide adequate immune stimulation for the candidate antigen, which is often a highly purified recombinant protein. Bacterial vectors from attenuated pathogenic or nonpathogenic backgrounds are commonly explored as suitable delivery systems for such antigens (Wang et al. 2013; Wells 2011). The advantage of these vectors is that many bacterial components are known to stimulate the innate immune system, a prerequisite to elicit strong and durable immune responses generated by the adaptive immune system (Mbow et al. 2010). In addition, the bacterial vectors present the antigen as a particle to the immune system, which is more advantageous than to use soluble antigens, especially if the vaccine is to be delivered through the mucosa such as in the oral or nasal cavities.

The noncovalent binding properties of LysM domains offer an opportunity to use non-GMO bacteria as vectors in vaccine development. This would eliminate one of the major regulatory hurdles associated with using vaccines employing a GMO carrier.

LysM_AcmA_ has been used extensively to construct non-GMO carriers, mainly in strategies to attach recombinant antigen-LysM fusions to cells of *L. lactis*, although the LysM_AcmA_ domain can bind to a broad range of Gram-positive bacteria (Bosma et al. 2006; Steen et al. 2003). Raha and co-workers used it for the display of VP1 epitopes of enterovirus type 71 on live nonrecombinant *L. lactis* for the development of an oral candidate vaccine against hand-foot-and-mouth disease (Raha et al. 2005; Varma et al. 2013). Likewise, Moeini et al. (2011) used LysM_AcmA_ to develop an oral chicken anemia virus vaccine based on live nonrecombinant *Lactobacillus acidophilus*. These oral vaccines raised detectable levels of antigen-specific serum antibodies in the animal models used (Table 3). While these vaccines are based on live bacteria, which may affect the stability and integrity of the antigens due to proteolytic activity by HtrA of the bacterial carrier, in other applications, a nonliving bacterial carrier (BLPs, see above) has been used. The BLPs exhibit an enhanced binding capacity for LysM domains and are currently employed in vaccine development. A comprehensive description of the BLP technology and its use in vaccines was recently published (Leenhouts 2013; van Braeckel-Budimir et al. 2013).

**Table 3:** Summary of LysM-based antigen display on nongenetically modified Gram-positive carriers for vaccine purposes

Carrier	Vaccine target	Antigen	Number of LysM sequences, N- or C-terminal fusion^a^	Model	Readout	Reference
*L. lactis*	Enterovirus type 71 (EV71)	VP1	C-1	Mice (oral)	Serum Ab response	Raha et al. (2005), Varma et al. (2013)
*L. acidophilus*	Chicken anemia virus	VP1	C-1 or C-2	Chickens (oral)	Serum Ab response; virus neutralization; Th1 cytokines (IL-2, IL-12, IFN-γ); splenocyte proliferation	Moeini et al. (2011)
*L. lactis* BLPs	*Plasmodium falciparum*	MSA2	C-3	Rabbit (i.n., oral)	Serum IgG response; mucosal IgA response	Ramasamy et al. (2006)
		VAR2CSA	C-2	Mouse (i.m.)	In progress	Expres2ion, Salati, and Mucosis, Eurostars project, unpublished
*L. lactis* BLPs	*Plasmodium berghei*	CSP	C-3	Mouse (i.m.)	Serum IgG response; IFN-γ-producing splenocytes; protection against challenge with *P. berghei* infected mosquitoes	Nganou-Makamdop et al. (2012)
*L. lactis* BLPs	*Streptococcus pneumoniae*	PpmA, SlrA, Iga1p	C-3	Mouse (i.n.)	Serum IgG response; protection against pulmonary challenge with *S. pneumoniae* D39	Audouy et al. (2006, 2007)
		PdBD, PspA, CbpA, PsaA	C-3	Mouse (i.n.)	Serum IgG response; protection against pulmonary challenge with *S. pneumoniae* TIGR4	Hermans, van Selm, Audouy, and Mucosis, unpublished
*L. lactis* BLPs	*Staphylococcus aureus*	LytM, Nuc, IsaA, IsdB	C-3	Mouse (s.c.)	Serum IgG response	van den Berg et al. (2011)
*L. lactis* BLPs	*Yersinia pestis*	LcrV	C-3	Mouse (i.n.)	Serum IgG, IgM, and IgA response; mucosal IgA; IgG and IgA Ab-secreting cell analysis; cytokine analysis; T cell proliferation; protection against i.v. challenge with *Y. pestis*	Ramirez et al. (2010)
*L. lactis* BLPs	*Shigella flexneri*	IpaB, IpaD	C-3	Mouse (i.n.)	Serum IgG response; mucosal IgA response; protection against pulmonary challenge with *S. flexneri* and *S. sonnei*	Pasetti, Picking, and Mucosis, unpublished
*L. lactis* BLPs	Influenza	HA	C-1	Mouse (i.n., i.m.)	Serum IgG response; serum HI titers; mucosal IgA response; protection against pulmonary challenge with H1N1 A/California/7/2009	De Haan, Rigter, Widjaja, Rottier, and Mucosis, unpublished
		M2e	C-3	Mouse (i.n.)	Serum IgG; protection against pulmonary challenge with H3N2 X47	Saelens and Leenhouts, unpublished
		NP	C-2	Mouse (i.n.)	Serum IgG; mucosal IgA; IFN-γ-producing splenocytes	Mucosis, unpublished
		NA	N-1	In vitro	Neuraminidase activity	De Haan, Rigter, Widjaja, Rottier, and Mucosis, unpublished
*L. lactis* BLPs	Respiratory syncytial virus (RSV)	F	C-1	Mouse (i.n.), cotton rat (i.n.)	Serum IgG; mucosal IgA; virus neutralization; viral lung titers after pulmonary challenge with RSV long	Rigter et al. (2013)

^a^Number and location of the LysM sequences in the fusion protein

BLPs still have the ability to stimulate the innate immune system (Ramirez et al. 2010) and were used in the development of bacterial, viral, and parasitic vaccines. An overview of antigen-to-LysM fusions that have been successfully attached to *L. lactis* BLPs is incorporated in Table 3. In general, BLP-based vaccines raise immune responses of a mixed Th1/Th2 nature and induce secretion of IgA at mucosal surfaces if the vaccine was administered through a mucosal route (Saluja et al. 2010). The immune responses were shown to be protective in animal challenge models for a subcutaneous (s.c.) malaria vaccine (Nganou-Makamdop et al. 2012), an intranasal (i.n.) multivalent pneumococcal vaccine (Audouy et al. 2007), an i.n. plague vaccine (Ramirez et al. 2010), an i.n. *Shigella* vaccine (Pasetti and Picking, personal communication), i.n. influenza vaccines (de Haan et al. 2012), and an i.n. respiratory syncytial virus (RSV) vaccine (Rigter et al. 2013). The antigens in the hemagglutinin (HA)-based influenza vaccine and fusion protein (F)-based RSV vaccine are each bound in their native homotrimeric conformation to the BLPs by combining a single LysM_AcmA_ sequence with an artificial multimerization domain. While proteins containing only one LysM_AcmA_ sequence are inefficient in PG binding, this function is restored by the addition of the leucine zipper-type GCN4 multimerization domain to the monomeric LysM sequence fusion protein (de Haan et al. 2012; Rigter et al. 2013). The GCN4 domain, placed between the antigen and a single LysM_AcmA_ sequence, effectively transforms the protein into a trimer that is capable of improved binding to BLPs while preventing monomeric proteins containing only a single LysM_AcmA_ sequence to be part of the vaccine as they bind less efficiently to the particles. In conclusion, efficient protein binding to BLPs (and possibly bacterial cells) via multiple LysM sequences does not require the motifs to be present in the same protein monomer. Using a similar approach, the influenza neuraminidase (NA) antigen was attached to BLPs in its native homotetrameric conformation (de Haan, personal communication). These examples show that complex proteins such as viral oligomeric proteins can also be successfully bound to BLPs.

BLPs offer as well the possibility to bind multiple antigens to a single carrier particle. One option is to bind antigens of different pathogens simultaneously to BLPs in order to create a combination vaccine. Another option is to bind multiple antigens from a single pathogen in order to create a multivalent vaccine that protects against a broad range of variants of the pathogen. The latter format has been examined by making a vaccine in which five different pneumococcal antigens were bound simultaneously to a single BLP carrier (van Roosmalen and Leenhouts, unpublished results). This pentavalent vaccine was compared with a vaccine in which the five antigens were individually bound to a BLP carrier after which the five BLP carriers were combined. Both types of vaccines were administered intranasally in mice and generated similar results in terms of serology and protection after challenge. Although the possibility to bind multiple antigens to a single carrier particle is easy and seems attractive, batch-to-batch consistency is a challenge, and therefore, it is more practical from a quality control point of view to combine different batches of BLPs with different antigens to generate combination or multivalent vaccines.

It was recently demonstrated that, in addition to the immune-stimulating effect of BLPs or live bacteria to which LysM-antigen fusions are bound, the LysM domain of the *L. monocytogenes* p60 endopeptidase itself has an effect on the immune system by activating natural killer (NK) cells (Schmidt et al. 2011). Whether this is a general characteristic for LysM domains or is restricted to *L. monocytogenes* LysM_p60_ remains to be established. Taken together, these data demonstrate that binding of antigens through one or more LysM sequences to nonliving, nonrecombinant bacterial particles can generate efficacious subunit vaccines.

## Other applications

Besides the vaccine applications described above, LysM domains are also being explored in additional potential health applications and for other purposes. Ravnikar et al. (2010) used a C-terminal fusion with LysM_AcmA_ to display a tumor necrosis factor alpha (TNF-α)-binding affibody on *L. lactis* cells. These could be effectively used to bind TNF-α. This setup was suggested to have potential in the treatment of inflammatory bowel disease.

LysM_AcmA_ has also been suggested to be of possible use to improve delivery of viable microorganisms to the intestinal tract (Tarahomjoo et al. 2008a, b, c). Employing a fusion of LysM_AcmA_ and the starch-binding domain of *S. bovis* α-amylase, adhesion of *L. casei* cells producing this chimeric protein to cornstarch was facilitated. The cornstarch-adhering *L. casei* survived better than wild-type *L. casei* or *L. casei* mixed with cornstarch during 2 h of incubation in simulated gastric juice. The authors speculate that this approach might be used for the protection of (probiotic) cells to the adverse gastric conditions, for instance by firmly encapsulating the bacteria in porous starch granules (Tarahomjoo et al. 2008c).

In a comparison of covalent versus noncovalent anchoring of the receptor-binding domain of the *E. coli* F18 fimbrial adhesin FedF to *L. lactis* NZ9000∆*htrA* cells, it was shown that noncovalent surface display using the *L. lactis* LysM_AcmA_ domain allowed the greatest amount of FedF protein to be surface-displayed compared to the amount of adhesin that could be covalently bound via the LPxTG_PrtP_ domain. A spacer consisting of the cell wall-spanning region of the PrtP protein was needed for proper cell surface display of FedF, a protein involved in adherence to porcine intestinal epithelial cells. Expression of the fusion protein in *L. lactis* NZ9000∆*htrA* allowed these cells to efficiently bind to the porcine cells (Lindholm et al. 2004).

Fredriksen et al. (2012) compared *L. plantarum* cells displaying the extracellular domain of invasin from the human pathogen *Yersinia pseudotuberculosis*, anchored to the cell surface either through membrane-anchoring domains or the LysM domain of a putative extracellular *L. plantarum* transglycosylase, for their capacity to activate monocytes in cell cultures. Enhanced activation of monocytes could improve the *L. plantarum* strains as delivery vehicle for vaccine antigens. In this case, the cells displaying invasin moieties that were membrane-anchored were most efficient in their monocyte activation capability.

Finally, the two N-terminal LysM sequences of the *B. anthracis* spore cortex lytic enzyme SleL have been shown to be responsible for localization of SleL to the coat or cortex of the endospore (Lambert et al. 2012). These authors suggested that spore decontamination methods might be developed by employing enzymes such as SleL; by externally adding SleL to spores, they might be triggered to initiate germination. The examples described above are most likely only the onset of possible other (health) application studies that will appear in the near future.

## Conclusions and future prospects

Since the first description of LysM-containing proteins and, subsequently, the discovery of their noncovalent binding to cells of Gram-positive bacteria, a host of publications have revealed various aspects of these proteins and their highly interesting LysM domains. First, it was shown that the LysM domain is widely spread in nature and is present not only in bacterial proteins but, most interestingly, also in many proteins of lower and higher eukaryotes, including mammals.

Research over the last two decades has revealed that the LysM sequence, which is present in LysM domains in one to multiple copies, is involved in binding of LysM-containing proteins to bacterial PG and, in some cases, also to chitin. Binding of the motif to PG is fast, robust, and strong. From an application point of view, the discovery that the LysM domain can be taken out of its natural context and be fused to other proteins was extremely important. Thus, it is now possible to attach, in principle, any protein or peptide of interest to PG-enveloped bacteria. Another key finding, and one opening up vast possibilities in the (mucosal) vaccine area, entails the increased and more stable binding of antigens fused to (a) LysM sequence(s) to acid-pretreated bacterial cells.

Using the LysM domain, binding of multiple different antigens and active enzymes has been achieved on living and nonliving cells, on spores, and to chitin. Removing cell wall components such as LTA, which hinder binding via the LysM domain, increases the strength of binding and the number of molecules that can be bound. This is most effectively realized by pretreating bacterial cells with trichloroacetic acid, which also eliminates proteases that could otherwise degrade the fusion proteins. Using LysM domains allows immobilizing purified fusion proteins on any Gram-positive bacterium in a non-GMO manner. The production of the LysM-containing proteins can best be performed in an autolysis- and proteolysis-negative protein expression host, such as *L. lactis ∆htrA∆acmA*. This strain lacks all extracellular proteolytic activity and does not autolyse, not even during the stationary phase of growth (Buist et al. 1995). As the LysM sequence specifically binds *N*-acetylglucosamine, one of the main components of the bacterial cell wall, the LysM sequence or a domain carrying multiple motifs could possibly be employed to detect Gram-positive (pathogenic) bacteria in different (bodily) fluids. One way to do this would be to fuse a LysM domain to a fluorescent protein, as was done for LysM_AcmA_ and the LysM domains of the staphylococcal proteins LytN and SleI (Table 3). In another setup, a biosensor could be developed to allow detecting minute levels of PG or microorganisms in solution. Owing to its ability to bind several substrates, LysM_AcmA_ in combination with another (cell wall) binding domain (binding, e.g., PG, cellulose and/or chitin) could be used for whole-cell immobilization. Such an approach might be useful in various industrial applications employing bacteria or chitin-containing fungal species to, for instance, avoid loss of cells.

The successful display of active enzymes using the LysM sequence could be extended by a display of a combination of enzymatic activities that together form a combined (metabolic) pathway or biosynthesis route. This method could potentially be extended or improved by making use of different noncovalent PG-binding domains (Table 1) in order to be able to more completely decorate carrier cells. Such a “multivalent” approach has already been tested for the display of multiple antigens on a single carrier.

In conclusion, a lot has been learned on the structure and function of the LysM domain, and this knowledge has been successfully explored in various potential applications. These studies have mainly been based on the use of bacterial LysM sequences in fusion proteins. Apart from the role of plant LysM sequences in plant-microbe signaling, virtually nothing is known about the function of LysM sequences in other eukaryotes. Much remains to be discovered in this area, especially with respect to possible future applications, e.g., in stimulating or steering beneficial eukaryote-microbe interactions or, possibly, identifying, typing, sensing, targeting, and/or combating plant and animal pathogens.
